# Exploring the Relationship Between Authentic Leadership and Nurses’ Caring Behavior: The Mediating Effect of Occupational Coping Self‐Efficacy

**DOI:** 10.1155/jonm/6142622

**Published:** 2026-02-27

**Authors:** Huiqi Chen, Yi Qiu, Chenglei Wu, Xiaoyun Li, Junxian Wu, Lin Li, Jing Yu, Jingru Song, Qin Shen

**Affiliations:** ^1^ School of Nursing, Zhejiang Chinese Medical University, Hangzhou, Zhejiang, China, zcmu.edu.cn; ^2^ Department of Respiratory and Critical Care Medicine, Jiangxi Provincial People’s Hospital, Nanchang, Jiangxi, China, jxsrmyy.cn

**Keywords:** authentic leadership, caring, clinical nurses, mediation analysis, occupational coping self-efficacy

## Abstract

**Background:**

Nurses’ caring behavior impacts patient outcomes. Studies have shown that authentic leadership and occupational coping self‐efficacy correlate with nurses’ caring behavior, but the mediating role of occupational coping self‐efficacy remains underexplored among Chinese nurses.

**Objective:**

This study aims to explore the relationship between authentic leadership and nurses’ caring behavior, as well as the mediating role of occupational coping self‐efficacy in this relationship.

**Methods:**

We recruited 436 nurses from 5 hospitals in Jiangxi and Zhejiang provinces, China, from November 2024 to January 2025. They completed online questionnaires, including general demographic information, the authentic leadership questionnaire, the occupational coping self‐efficacy questionnaire, and the caring behavior scale. A total of 436 questionnaires were collected, of which 418 were valid and included in the analysis. Structural equation modeling (SEM) and bootstrapping procedures were used to examine the mediating role of occupational coping self‐efficacy.

**Results:**

Clinical nurses scored 72.00 (64.00, 80.00) for authentic leadership, 37.00 (34.00, 43.00) for occupational coping self‐efficacy, and 137.00 (120.00, 144.00) for caring behavior. Authentic leadership was positively correlated with occupational coping self‐efficacy and caring behavior (*r* = 0.652, *p* < 0.001; *r* = 0.634, *p* < 0.001), and caring behavior was positively correlated with occupational coping self‐efficacy (*r* = 0.748, *p* < 0.001). The direct effect of authentic leadership on the caring behavior of nurses was not significant (*β* = 0.061, *p* = 0.448, 95% CI: −0.091–0.205); occupational coping self‐efficacy exerted a total mediating role between authentic leadership and caring behavior (*β* = 0.441, *p* < 0.001, 95%CI: 0.341–0.567).

**Conclusion:**

The scores for caring behavior, occupational coping self‐efficacy, and authentic leadership were all at a moderate to high level. Both authentic leadership and occupational coping self‐efficacy were crucial in promoting nurses’ caring behavior, with occupational coping self‐efficacy fully mediating the effect of authentic leadership on caring behavior. However, due to the cross‐sectional design of this study, causal relationships between the variables cannot be established. Future research should employ longitudinal designs to further investigate these relationships.

**Implications for Nursing Management:**

Nursing administrators should focus on the impact of occupational coping self‐efficacy and authentic leadership on caring behavior. In addition to authentic leadership training, situational simulation exercises and peer support programs can be used to increase nurses’ occupational coping self‐efficacy, which will help to promote caring behavior and enhance the quality of care.

## 1. Introduction

Caring behavior is a core component of nursing care. Conceptually, caring behavior includes two essential elements: one is instrumental caring behavior, which is related to technical and physical behavior, and the other is emotional caring behavior, which provides for expressing kindness and conveying confidence to patients [1]. Research has shown that providing sustained, high‐level care can increase patient satisfaction, reduce adverse events, and improve the overall quality of care [2, 3]. In the Chinese clinical setting, nursing staffing shortages have led to an overburdening of nurses’ daily care duties. This may result in reduced attention to patients’ psychological and emotional needs. For example, nurses may lack the time to address patients’ questions or provide emotional reassurance [4]. In order to meet the demand for high‐quality health care, it is particularly important to systematically identify and elucidate the factors influencing nurses’ caring behavior and their underlying mechanisms.

The caring behavior of nurses is influenced by multiple factors, including individual‐level factors such as work experience and job satisfaction, as well as organizational‐level factors such as the work environment [5, 6]. Leadership, as a core component of the work environment, is of great interest because of its significant influence on individual behaviors [7–9]. Previous research has shown that different leadership styles can influence nurse behavior through different psychological pathways [10, 11]. Among the common transformational, inclusive, and authentic leadership, the first two mainly enhance psychological empowerment through vision communication and intellectual stimulation and psychological safety through open communication and respect for differences, respectively [8, 10]. Authentic leadership is a style of leadership behavior that promotes positive mental abilities, creates a positive ethical climate, and is effective in promoting positive self‐development in followers. It centers on self‐awareness, relational transparency, internalized moral perspective, and balanced processing [12]. Compared to other leadership styles, authentic leadership is rooted in humanistic values and aligns more closely with the ethically sensitive and highly interpersonal nature of nursing work [13–15]. It has been demonstrated to be positively correlated with nurses’ caring behavior [16], but the specific pathways through which authentic leadership influences nurses’ caring behavior remain unclear.

Occupational coping self‐efficacy, as a key mediating variable linking organizational environment and individual behavior, has garnered increasing attention. Occupational coping self‐efficacy refers to nurses’ confidence in their ability to cope with the demands of work [17]. In the nursing field, it is crucial for improving nurses’ mental health, alleviating occupational burnout, and reducing implicit absenteeism [18–20] and is positively correlated with nurses’ caring behavior [21]. Specifically, nurses with high occupational coping self‐efficacy tend to exhibit stronger competence expectations and goal commitment and lower emotional exhaustion; these characteristics collectively enhance the level of work embeddedness, thereby consistently delivering high‐quality care across a range of tasks, including communication, comforting, and health education [22, 23]. In addition, there is a positive correlation between authentic leadership and nurses’ occupational coping self‐efficacy. Authentic leaders are able to create a supportive work environment and increase nurses’ confidence in coping with work challenges by presenting their true selves and constructing positive and sincere relationships with their superiors and subordinates, thus effectively enhancing nurses’ occupational coping self‐efficacy, a critical psychological resource [24, 25].

Although the results of previous studies have confirmed the two‐by‐two correlation between authentic leadership, occupational coping self‐efficacy, and caring behavior in nurses, however, the mediating role of occupational coping self‐efficacy has not been fully explored, especially since there is limited empirical research on clinical nurses in China. This has constrained our understanding of the formation mechanisms of caring behavior in clinical settings and their potential mediating pathways. To address this gap, this study employs Bandura’s social cognitive theory as its theoretical framework [26], which emphasizes that an individual’s behavior is determined by the interaction among the individual, the behavior, and the environment and states that an individual’s behavior is not only influenced by the external environment but also regulated by internal cognitive processes. Self‐efficacy, as an internal cognitive factor, serves as the core mediator linking situational cues to behavioral outcomes, significantly influencing the initiation, engagement, and persistence of behavior. Its primary sources include mastery experiences, vicarious experiences, verbal persuasion, and an individual’s emotional and physiological state [27]. In nursing organizational settings, authentic leadership serves as a critical environmental factor. Through management practices such as relational transparency, value alignment, participatory decision‐making, and procedural justice, it creates the prerequisites for enhancing nurses’ occupational coping self‐efficacy. Specifically, authentic leadership enables nurses to accumulate mastery experiences through empowerment and challenging opportunities, fosters vicarious learning through high‐quality modeling, provides timely affirmation as verbal encouragement, and cultivates psychological safety to optimize emotional states. The resulting heightened self‐efficacy empowers nurses to confidently believe in their capacity to perform caring behavior and other positive professional actions when confronting heavy workloads and complex patient conditions, thereby driving the initiation and maintenance of such behaviors.

Therefore, based on the aforementioned theoretical and empirical evidence, this study aims to examine the impact of authentic leadership on nurses’ caring behavior, with a particular focus on revealing the mediating role of occupational coping self‐efficacy in this relationship. This research seeks to provide theoretical foundations and practical insights for systematically enhancing the caring competency of clinical nurses.

The specific research hypotheses are as follows:

**Hypothesis 1.**

Authentic leadership has a direct positive impact on nurses’ caring behavior.

**Hypothesis 2.**

Authentic leadership has a direct positive impact on occupational coping self‐efficacy.

**Hypothesis 3.**

Occupational coping self‐efficacy has a direct positive impact on nurses’ caring behavior.

**Hypothesis 4.**

Occupational coping self‐efficacy mediates the relationship between authentic leadership and nurses’ caring behavior.

### 1.1. Objective

This study aims to (1) investigate the current status of authentic leadership, occupational coping self‐efficacy, and caring behavior in nurses; (2) explore the interrelationships between authentic leadership, occupational coping self‐efficacy, and caring behavior in nurses; (3) and analyze the mediating role of occupational coping self‐efficacy between authentic leadership and caring behavior.

## 2. Methods

### 2.1. Study Design

We conducted a cross‐sectional study on nurses, adhering to STROBE guidelines for observational studies [28].

### 2.2. Setting and Participants

From November 2024 to January 2025, clinical nurses from five tertiary public hospitals in Jiangxi and Zhejiang provinces were selected for this study using a convenience sampling approach. Nurses had to meet the following inclusion criteria: (a) having worked for a year or more in the current hospital, (b) being presently employed in a hospital as a registered nurse, and (c) being willing to participate in the study. Exclusion criteria were: (a) nurses who were not on duty during the study period for various reasons and (b) those who had mental illnesses. Nurses who withdrew from the study or did not complete the questionnaire were excluded from the analysis. Usually, the sample size is 5–10 times the number of entries and 20% of invalid questionnaires are taken into account, so at least a sample size of 354 is needed. A total of 436 nurses were invited to participate, and 436 questionnaires were collected. After excluding low‐quality questionnaires with patterned or identical responses, 418 valid questionnaires were included for analysis, resulting in an effective response rate of 95.87%. No missing data were identified.

### 2.3. Measures

#### 2.3.1. General Information Questionnaire

It included 10 closed‐ended questions designed by the researcher based on the relevant literature, such as gender, age, years of working, education level, marital status, professional title, employment status, frequency of night shifts, humanistic caring training, and job satisfaction.

#### 2.3.2. Authentic Leadership Questionnaire (ALQ)

ALQ was developed by Walumbwa in 2008 [12]. It was translated and culturally adapted by Han to make the ALQ more appropriate for Chinese nurses [29], which consists of four dimensions: relational transparency (5 items), morals and ethics (4 items), balanced processing (3 items), and self‐awareness (4 items), with a total of 16 items. The scale was scored on a 5‐point Likert scale, with scores ranging from 1 to 5 on a scale from “strongly disagree” to “strongly agree” and the total score ranging from 16 to 80, with higher scores indicating higher levels of leader authenticity perceived by nurses. This scale has been applied in empirical research within the Chinese context and has demonstrated good reliability and validity. Structural validity indices: χ^2^/df = 1.830, CFI = 0.950, RMSEA = 0.060. The Cronbach’s *α* coefficient of this scale is 0.850. The Cronbach’s *α* coefficient of this scale in this study is 0.978.

#### 2.3.3. Occupational Coping Self‐Efficacy Scale for Nurse (OCSE‐N)

OCSE‐N was developed by Pisanti in 2008 [17]. It was translated and culturally adapted by Zhai [30] and consists of 9 items in two dimensions: occupational burden and relationship difficulties. The scale was scored on a 5‐point Likert scale, with scores ranging from 1 to 5 on a scale from “not able to cope easily” to “completely able to cope easily” and the total score ranging from 9 to 45. The higher the score, the higher the self‐efficacy of nurse’s occupational coping. The Chinese version of this scale demonstrated good reliability and validity. Structural validity indices: χ^2^/df = 1.452, GFI = 0.987, AGFI = 0.977, RMR = 0.035, NFR = 0.977, RFI = 0.969. The Cronbach’s *α* coefficient of this scale is 0.882. The Cronbach’s *α* coefficient of the scale in this study is 0.941.

#### 2.3.4. Caring Behavior Inventory (CBI)

CBI was developed by Wolf [31]; it was translated and culturally adapted by Da [32], which was used to evaluate the implementation of nurses’ caring behavior. The scale consists of three dimensions: respect and connection (10 items), knowledge and skills (5 items), and support and reassurance (9 items), with a total of 24 items. The Likert 6‐point scale was used, with scores ranging from 1 to 6, from “never” to “always,” and the total scores ranging from 24 to 144; the higher the score, the more caring the nurse was. The Chinese version of the caring behavior scale demonstrated good reliability and validity. Structural validity indices: χ^2^/df = 2.421, GFI = 0.908, CFI = 0.921, RMSEA = 0.035. The Cronbach’s *α* coefficient of this scale is 0.959. The Cronbach’s ɑ coefficient of the scale in this study was 0.981.

### 2.4. Data Collection

Before data collection, the researchers obtained approval from the nursing managers of the selected departments and visited each unit in person to meet with all potential participants. This allowed for face‐to‐face verification of each nurse’s registration status and inclusion eligibility. After being fully informed of the study purpose, procedures, and confidentiality principles, participants voluntarily signed an electronic informed consent form. The online questionnaire link was then distributed via platforms such as WeChat, and participants independently completed and submitted the survey using their personal mobile devices. The survey was conducted anonymously using Wenjuanxing, an online survey tool widely used in China that supports encrypted data collection, anonymous submission, and secure data export. To ensure data integrity, all questions are mandatory. When submitting the questionnaire, the system will automatically flag any incomplete entries and prevent submission until all questions are answered. To prevent duplicate responses, only one questionnaire may be submitted per IP address.

### 2.5. Statistical Analysis

SPSS 25.0 and AMOS 24.0 were used for data analysis. General information is described using frequency and percentage, while continuous variables with skewed distributions, determined by normality tests, were presented as medians and interquartile ranges (IQRs), and Spearman’s rank correlation was used to analyze the correlation between the variables. To assess potential common method bias, Harman’s single‐factor test was performed by subjecting all items to unrotated principal component analysis. A variance explained by the first factor of less than 40% was considered indicative of negligible common method bias. Structural equation modeling (SEM) was used to confirm whether occupational coping self‐efficacy mediated the relationship between authentic leadership and nurses’ caring behavior. A 5000‐sample bootstrapping procedure with a 95% confidence interval (CI) was used to estimate and test the mediating role [33]. The *p* value was less than 0.05, which was considered significant. All significant tests were two‐tailed, with a 5% level of significance.

### 2.6. Ethical Considerations

This study was approved by the Medical Ethics Committee of Zhejiang Chinese Medicine University (Approval No. 20241108‐3). During the survey process, strict adherence was maintained to the principles of voluntary participation and anonymous completion. Survey participants retained the right to withdraw from the study at any time. Collected data were used only for academic research purposes and were not disclosed to unauthorized individuals.

## 3. Results

### 3.1. Testing for Common Method Bias

The Harman’s single‐factor test was used to detect potential common method bias between the questionnaires. The factor analysis results indicated that five factors with eigenvalues greater than 1 could be extracted, and the first factor explained 25.87% of the variance, which meets the threshold of < 40%. Therefore, it can be concluded that there is no significant common method bias in this study.

### 3.2. Nurses’ Basic Characteristics and Scores of Authentic Leadership, Occupational Coping Self‐Efficacy, and Caring Behavior

A total of 436 nurses were invited to participate in this study, with all 436 completing the questionnaire. After excluding low‐quality questionnaires with repetitive or identical responses, 418 valid questionnaires were ultimately included in the analysis. The nurses’ basic characteristics are shown in Table 1. The scores of authentic leadership, occupational coping self‐efficacy, and caring behavior and their subdomain scores are shown in Table 2.

**TABLE 1:** Basic characteristics of the nurses (*n* = 418).

Variables	Categories	*n*	%
Gender	Men	18	4.3
	Women	400	95.7

Age (years)	≤ 25	61	14.6
	26–30	102	24.4
	31–40	174	41.6
	≥ 41	81	19.4

Years of working	1–5	111	26.5
	6–10	68	16.3
	11–20	168	40.2
	≥ 21	71	17.0

Education level	Junior college degree or below	54	12.9
	Bachelor’s degree	358	85.6
	Master’s degree or above	6	1.5

Marital status	Single	140	33.5
	Married	268	64.1
	Divorced or widowed	10	2.4

Professional title	Nurse	67	16.0
	Nurse practitioner	128	30.6
	Nurse in charge	177	42.4
	Associate professor of nursing or above	46	11.0

Employment status	Permanent	190	45.5
	Supernumeraries	228	54.5

Frequency of night shifts	No night work	133	31.8
	1–3 times per month	44	10.5
	≥ 4 times per month	241	57.7

Humanistic caring training	Yes	224	53.6
	No	194	46.4

Job satisfaction	Dissatisfied	18	4.3
	Average	137	32.8
	Satisfied	263	62.9

**TABLE 2:** The total and subdomain scores of AL, OCSE, and CB [*n* = 418, *M* (*p*_25_, *p*_75_)].

Variables and subdomains	Median	Average	Average score index (%)
AL	72.00 (64.00, 80.00)	4.50 (4.00, 5.00)	90.00
Self‐awareness	19.00 (16.00, 20.00)	4.75 (4.00, 5.00)	95.00
Morals and ethics	18.00 (16.00, 20.00)	4.50 (4.00, 5.00)	90.00
Relational transparency	23.00 (20.00, 25.00)	4.60 (4.00, 5.00)	92.00
Balanced processing	13.00 (12.00, 15.00)	4.33 (4.00, 5.00)	86.60
OCSE	37.00 (34.00, 43.00)	4.11 (3.78, 4.78)	82.20
Relationship difficulties	13.00 (12.00, 15.00)	4.33 (4.00, 5.00)	86.60
Occupational burden	24.00 (22.00, 29.00)	4.00 (3.67, 4.83)	80.00
CB	137.00 (120.00, 144.00)	5.71 (5.00, 6.00)	95.17
Respect and connection	55.00 (49.00, 60.00)	5.50 (4.90, 6.00)	91.67
Knowledge and skills	30.00 (25.00, 30.00)	6.00 (5.00, 6.00)	100.00
Support and reassurance	54.00 (45.00, 54.00)	6.00 (5.00, 6.00)	100.00

Abbreviations: AL, authentic leadership; CB, caring behavior; OCSE, occupational coping self‐efficacy.

### 3.3. Correlation Among Authentic Leadership, Occupational Coping Self‐Efficacy, and Caring Behavior

Spearman rank correlation analyses showed that authentic leadership was positively correlated with both occupational coping self‐efficacy and caring behavior (*r* = 0.652, *p* < 0.001; *r* = 0.634, *p* < 0.001) and that occupational coping self‐efficacy was positively correlated with nurses’ caring behavior (*r* = 0.748, *p* < 0.001) (Table 3)

**TABLE 3:** Statistical description and correlation matrix results for study variables (*n* = 418, *r*).

	AL	SA	ME	RT	BP	OCSE	RD	OB	CB	RC	RS	SR
AL	1											
SA	0.929^∗∗^	1										
ME	0.945^∗∗^	0.862^∗∗^	1									
RT	0.945^∗∗^	0.869^∗∗^	0.871^∗∗^	1								
BP	0.928^∗∗^	0.885^∗∗^	0.891^∗∗^	0.821^∗∗^	1							
OCSE	0.652^∗∗^	0.636^∗∗^	0.609^∗∗^	0.603^∗∗^	0.616^∗∗^	1						
RD	0.671^∗∗^	0.644^∗∗^	0.635^∗∗^	0.631^∗∗^	0.634^∗∗^	0.904^∗∗^	1					
OB	0.593^∗∗^	0.579^∗∗^	0.552^∗∗^	0.546^∗∗^	0.561^∗∗^	0.973^∗∗^	0.799^∗∗^	1				
CB	0.634^∗∗^	0.630^∗∗^	0.585^∗∗^	0.590^∗∗^	0.603^∗∗^	0.748^∗∗^	0.696^∗∗^	0.723^∗∗^	1			
RC	0.614^∗∗^	0.609^∗∗^	0.561^∗∗^	0.572^∗∗^	0.583^∗∗^	0.747^∗∗^	0.692^∗∗^	0.722^∗∗^	0.974^∗∗^	1		
RS	0.558^∗∗^	0.549^∗∗^	0.537^∗∗^	0.530^∗∗^	0.520^∗∗^	0.653^∗∗^	0.628^∗∗^	0.627^∗∗^	0.892^∗∗^	0.832^∗∗^	1	
SR	0.563^∗∗^	0.564^∗∗^	0.521^∗∗^	0.527^∗∗^	0.529^∗∗^	0.645^∗∗^	0.615^∗∗^	0.616^∗∗^	0.912^∗∗^	0.839^∗∗^	0.873^∗∗^	1

Abbreviations: AL, authentic leadership; BP, balanced procession; CB, caring behavior; ME, morals and ethics; OB, occupational burden; OCSE, occupational coping self‐efficacy; RC, respect and connection; RD, relationship difficulties; RS, knowledge and skills; RT, relational transparency; SA, self‐awareness; SR, support and reassurance.

^∗∗^ *p* < 0.001.

### 3.4. SEM

**FIGURE 1:**
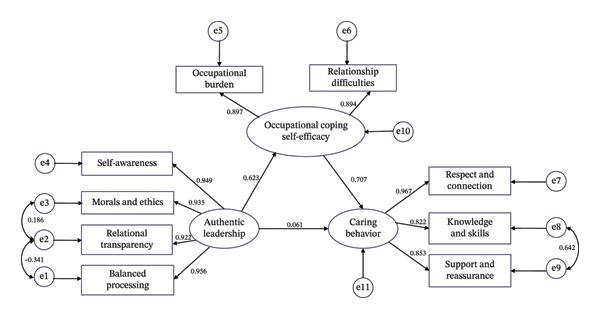
The final model of caring behavior (with standardized regression coefficients).

The SEM was constructed with authentic leadership as the independent variable, caring behavior as the dependent variable, and occupational coping self‐efficacy as the mediating variable. The fit indices were not sufficiently good after the initial model was run; the model was further improved by considering MIs and correlation among variables [34]; that is, each modification was tested for correlation before iterative incorporation, and the model fit improved with each iteration. The MIS considered in the study was from the same scale, so it was theoretically reasonable to have a high covariance. By correlating theoretically related error terms based on MIS, the final model improved substantially (RMSEA = 0.065, *χ* ^2^/df = 2.767, GFI = 0.970, CFI = 0.991, NFI = 0.986, TLI = 0.985), as shown in Figure 1.

The path analysis results of the SEM showed that authentic leadership had a positive impact on occupational coping self‐efficacy among nurses (standardized coefficient *β = *0.623, *p* <  0.001, 95%CI: 0.508–0.714). The higher the level of head nurses’ authentic leadership perceived by nurses, the higher the level of occupational coping self‐efficacy among nurses. Occupational coping self‐efficacy also had a positive impact on nurses’ caring behavior (standardized coefficient *β = *0.707, *p* <  0.001, 95% CI: 0.573–0.838); the higher the level of occupational coping self‐efficacy among nurses, the higher their level of caring behavior. However, the predictive effect of authentic leadership on nurses’ caring behavior was not significant (standardized coefficient *β* = 0.061, *p* = 0.448 [> 0.05, not significant]), which suggests that the direct impact of authentic leadership on nurses’ caring behavior is not significant.

We used the bias‐corrected bootstrap 95% CI based on 5000 bootstrap samples to test the mediating effect. As shown in Table 4, the deviation CI for the indirect effect path of authentic leadership on nurses’ caring behavior was 0.341–0.567 (standardized coefficient *β* = 0.441, *p* < 0.001), and the CI did not include 0; this showed that the mediating effect of occupational coping self‐efficacy among nurses was significant. Conversely, the deviation CI of the direct impact path of authentic leadership on nurses’ caring behavior was −0.091–0.205, and the CI included 0. This indicated that there was no direct effect of authentic leadership on nurses’ caring behavior. Consequently, occupational coping self‐efficacy had a complete mediating effect between authentic leadership and nurses’ caring behavior.

**TABLE 4:** Results for the total, indirect, and direct effects of authentic leadership on caring behavior with occupational coping self‐efficacy as a mediator (*n* = 418).

Effect	Estimate (*β*)	SE	95% CI		*p* value
			Lower	Upper	
Direct effect	0.061	0.075	−0.091	0.205	0.448
Indirect effect	0.441	0.057	0.341	0.567	< 0.001
Total effect	0.502	0.057	0.391	0.610	0.001

Abbreviations: CI, confidence interval; SE, standard error.

## 4. Discussion

### 4.1. Current Status of Authentic Leadership, Occupational Coping Self‐Efficacy, and Nurses’ Caring Behavior

The results of this study showed that the overall level of authentic leadership among nurses in this sample was at a moderately high level, higher than that reported by Teo et al. [35] among Australian nurses. This difference may be related to cultural context and sample composition. First, in terms of cultural context, Confucianism has a profound influence in China. It has long emphasized “self‐cultivation” and “self‐reflection,” advocating self‐discipline guided by virtue and role‐based ethics [36]. This orientation leads managers to place greater emphasis on demonstrating self‐awareness and relational transparency and to stress fairness and moderation in organizational governance, thereby enhancing nurses’ perceived scores in dimensions such as self‐awareness. Second, regarding sample composition, nurses with ≥ 10 years of work experience accounted for a higher proportion in this study (57.1%). Senior nurses have a greater sense of competence in their work and tend to be regarded as the backbone of the department and are more likely to establish high‐quality leader–member exchange relationships with their managers [37]. Therefore, they tend to have more positive perceptions of leaders’ transparency, integrity, and moral consistency [38]. Nursing managers should strengthen authentic leadership attributes such as self‐awareness and relational transparency to foster a high‐trust departmental culture. In addition, it is essential to value and retain experienced nurses by providing opportunities for participatory decision‐making and empowerment. Through high‐quality leader–member exchanges, these measures can enhance the team’s positive perceptions of authentic leadership and further promote caring practices.

The results of this study indicated that the overall level of occupational coping self‐efficacy among clinical nurses was moderately high, which was higher than the level reported by Lin et al. among ICU nurses [19]. This difference may be attributed to variations in the work environment. ICU nurses care for critically ill patients whose conditions change rapidly, and they are required to perform highly intensive and technically demanding nursing tasks while also coping with the psychological stress associated with end‐of‐life care. Prolonged exposure to such high workload environments can lead to continuous depletion of physical and psychological resources, resulting in elevated levels of job burnout and the development of more negative self‐cognitions [39]. These negative perceptions may cause nurses to underestimate their own coping abilities when faced with complex nursing tasks, thereby undermining their confidence in managing work‐related challenges and ultimately constraining the development of occupational coping self‐efficacy. In contrast, most participants in the present study worked in general wards, where patients’ conditions are usually more stable and nursing tasks are more predictable and structured. In such environments, nurses can efficiently complete nursing tasks by following well‐established procedures and continuously accumulate coping experience through daily practice. This sense of professional control and positive feedback from their work experiences contributes to stronger confidence in their abilities and enhances their occupational coping self‐efficacy. Therefore, it is recommended that nursing managers organize thematic seminars or establish working groups to develop and implement targeted stress management interventions aimed at improving nurses’ ability to cope with occupational stress [40]. Meanwhile, adequate staffing and optimized nurse–patient ratios should be ensured to effectively reduce nurses’ workload and further strengthen their occupational coping self‐efficacy.

The results of this study showed that the overall score of caring behavior among clinical nurses was at a moderately high level, consistent with the findings of Sedighi et al. [41]. Among the dimensions, the knowledge and skill dimensions scored relatively high, which may be related to the fact that the current nursing clinical training system focuses more on the training and assessment of standardized operational procedures. In contrast, the respect and connectedness dimensions scored comparatively lower. One possible explanation is that all participants in this study were from tertiary hospitals, where nurses tend to focus more on completing treatment procedures and ensuring patient safety, leaving limited time and energy for in‐depth emotional communication and relationship building [42]. In addition, nurses are often exposed to high‐stress environments characterized by pain, grief, and death, which may lead to emotional exhaustion and compassion fatigue and consequently hinder the implementation of affective caring behavior [43]. It is recommended that nursing managers strengthen systematic training in nurses’ emotional communication skills, pay close attention to their emotional well‐being, provide timely psychological support, and foster a positive and healthy organizational climate to further promote the expression of caring behavior.

### 4.2. The Inter‐Relationship Between Authentic Leadership, Occupational Coping Self‐Efficacy, and Caring Behavior Among Nurses

The results of the study confirmed that there is a positive correlation between authentic leadership and nurses’ caring behavior, supporting Hypothesis 1, which is consistent with previous research [16]. Social cognitive theory indicates that individual behavior is influenced by the external environment. When nursing managers act in an authentic leadership style, demonstrating consistency between words and actions, maintaining transparency, and ensuring equitable participation, nurses often perceive them as trustworthy role models. Through observation, imitation, and gradual internalization of managerial behaviors, nurses transform caring principles such as “respect, empathy, and support” into stable daily practices. Moreover, authentic leadership can foster and deepen nurses’ caring behavior across multiple dimensions. On one hand, open communication and relational transparency help build trust and high‐quality leader–member relationships, enhancing nurses’ job satisfaction and enabling them to become more engaged in care while paying closer attention to patients’ emotional needs [44]. On the other hand, leaders’ ethical consistency and people‐centered values reinforce nurses’ commitment to patient‐centered care, allowing kindness and respect to flow more naturally into their interactions [45]. Simultaneously, the self‐awareness and rational thinking advocated by authentic leadership help nurses balance clinical protocols, evidence‐based practices, and patient preferences in complex situations, thereby improving the timeliness and appropriateness of care delivery [46]. Therefore, the cultivation of authentic leadership among nursing managers should be strengthened. For instance, incorporating authentic leadership workshops or case discussions into management training can help head nurses learn to manage teams with honesty, respect, and fairness, thereby becoming credible role models. Concurrently, nursing managers should actively demonstrate caring behavior during routine ward rounds and team interactions to foster a culture of compassion within the organization. Through these measures, nurses can internalize the concept of care subtly, enhance their awareness of humanistic care, and ultimately elevate the overall quality of nursing services.

The results of the study showed a positive correlation between authentic leadership and occupational coping self‐efficacy, supporting Hypothesis 2, which is consistent with previous research [24]. According to Bandura’s social cognitive theory, authentic leadership, as an external environmental factor, shapes and enhances nurses’ self‐efficacy. Specifically, authentic leadership facilitates the decomposition of complex tasks into actionable phased objectives through empowering practices, supplemented by process guidance and constructive feedback. This approach fosters the formation of transferable “success scripts,” shifting efficacy judgments from fixed abilities to controllable strategies. Nurses thereby accumulate mastery experiences by progressively achieving incremental successes [47]. At the same time, the stable values and coping strategies they demonstrate when navigating ethical dilemmas and interpersonal pressures also provide nurses with alternative experiences to draw upon [48]. Moreover, leaders’ timely, specific, and task‐oriented positive feedback constitutes effective verbal encouragement, continuously reinforcing nurses’ positive self‐assessment of their capabilities, and their creation of an open, transparent, and supportive environment effectively buffers nurses’ work‐related stress and anxiety, thereby optimizing their emotional and physiological states [13]. Authentic leadership synergistically promotes the integration of self‐efficacy information and the formation of beliefs among nurses through these multidimensional practices, ultimately enhancing their occupational coping self‐efficacy. Nursing managers should establish institutionalized participation mechanisms to systematically embed frontline nurses’ decision‐making involvement in process optimization and quality improvement initiatives. Through progressive task delegation and phased goal‐setting, supplemented by structured training programs, resource allocation, and process‐based feedback, nurses can accumulate successful experiences within manageable challenges. This approach effectively consolidates their mastery experiences and promotes steady enhancement of their occupational coping self‐efficacy.

The results of the study also showed a positive correlation between occupational coping self‐efficacy and nurses’ caring behavior, supporting Hypothesis 3. This is consistent with previous research [21], which indicates that the higher the level of nurses’ occupational coping self‐efficacy, the more effectively they can practice caring behavior in their daily nursing work. Social cognitive theory posits that self‐efficacy serves as the foundation of individual motivation and influences outcome behavior and expectations [49]. Nurses with high occupational self‐efficacy possess greater confidence in their professional capabilities, firmly believing they can proactively address tasks and challenges encountered in their work. This positive self‐perception fosters higher levels of intrinsic motivation and responsibility, driving nurses to pursue high‐quality care in their practice [27]. Since caring behavior is a key manifestation of high‐quality nursing, nurses with high self‐efficacy are more inclined to invest emotional labor in patient interactions [22]. They demonstrate greater empathy, patience, and attentive care, enabling them to keenly discern patient needs and provide appropriate responses. This transforms their pursuit of high‐quality care into consistent, observable acts of caring behavior. Nursing managers can utilize scenario‐based training (such as conflict communication) to help nurses gain experience and strengthen their self‐efficacy beliefs. This facilitates a transition from psychological empowerment to behavioral improvement, ultimately enhancing the level of caring behavior.

### 4.3. The Mediating Effect of Occupational Coping Self‐Efficacy Between Authentic Leadership and Nurses’ Caring Behavior

This study found that the direct effect between authentic leadership and nurses’ caring behavior was not significant. Possible reasons for this include the following: (1) Guided by the core concept of “environment‐individual‐behavior” dynamic interaction in social cognitive theory, this study posits authentic leadership as a pivotal external environmental factor. It enhances nurses’ occupational coping self‐efficacy by fostering a supportive climate and providing empowering practices and resource support, thereby strengthening the intrinsic motivation for caring behavior. When this internal cognitive mechanism is sufficiently activated and dominates behavioral regulation, the direct influence of external leadership behaviors on caring conduct may correspondingly diminish. (2) The direct effect of authentic leadership on caring behavior may only be significant under specific conditions or within certain subgroups. As this study did not conduct subgroup analyses, potential differences between these subgroups may have been obscured in the overall analysis, where effects could average out or cancel each other, thereby masking localized significant relationships. (3) The direct influence of authentic leadership may require a certain period to accumulate before becoming evident. However, this study adopted a cross‐sectional design, which may not adequately capture this dynamic developmental process. In summary, researchers should interpret the present findings with caution. It is recommended that future studies employ longitudinal tracking and subgroup designs while controlling for confounding variables to further validate the mechanism through which authentic leadership influences nurses’ caring behavior.

Occupational coping self‐efficacy fully mediated the relationship between authentic leadership and nurses’ caring behavior, supporting Hypothesis 4. This indicates that authentic leadership indirectly influences nurses’ caring behavior through occupational coping self‐efficacy. According to the social cognitive theory, an individual’s behavior is influenced by both the external environment and internal cognition, and the external environment also influences an individual’s internal cognition [26]. In the unique context of nursing care, genuine caring behavior is endogenous and self‐motivated in nature, making it difficult to achieve through standardized procedures or external enforcement. It is precisely in such contexts that authentic leadership demonstrates its distinctive value. By championing openness and transparency, encouraging subordinate participation in decision‐making, and acting with sincerity and moral integrity, authentic leaders foster a positive and trusting organizational atmosphere. This environment facilitates the activation and development of followers’ internal resources, thereby enhancing nurses’ occupational coping self‐efficacy [24]. Nurses with high occupational coping self‐efficacy tended to adopt more positive coping styles in the face of stress and challenges at work, such as seeking support and resources. Having sufficient resources to meet the demands of the job will help encourage self‐improvement and positive behaviors at work [22]. Specifically, this is reflected in nurses’ ability to go beyond mechanical task execution and, driven by professional confidence, actively mobilize both cognitive and emotional resources to deliver truly individualized and humanized clinical care. Therefore, authentic leadership does not promote caring behavior through direct instruction, but rather by shaping a supportive environment that enhances nurses’ occupational coping self‐efficacy—an essential form of psychological capital—thereby indirectly and profoundly inspiring genuine caring behavior. This new insight provides empirical support for Bandura’s social cognitive theory.

### 4.4. Limitations

This study has several strengths. First, it employed validated measurement tools with confirmed good reliability and validity. Second, the research examined the mediating role of occupational coping self‐efficacy in the relationship between authentic leadership and nurses’ caring behavior, providing empirical support for developing intervention strategies to enhance the level of caring behavior among nurses.

Nonetheless, several limitations should be considered when interpreting the results: (1) The use of a convenience sampling method with all participants recruited from Chinese tertiary hospitals may limit the generalizability of the findings across different cultural contexts. Thus, further validation in diverse settings is warranted. (2) This study estimated sample size based on rule of thumb, a method commonly used in practice but lacking rigorous statistical power analysis. Nevertheless, the final collected effective sample size far exceeded the estimated requirement, which to some extent enhanced the study’s statistical power and reduced the risk of type II errors. Future research should employ more precise power analysis (e.g., using G∗Power software) during the design phase to improve methodological rigor. (3) The questionnaire employed a self‐report method and was distributed via head nurses and WeChat platforms. Although participation was voluntary and anonymous, responses may still be influenced by social expectations or concerns about self‐image, potentially introducing response bias. Future studies may consider utilizing multisource data (such as peer‐rated reports) to gather information. (4) In constructing the structural model, this study did not statistically control for demographic variables such as age, shift type, and work experience, nor did it examine their moderating effects. Consequently, the relationships among the primary variables may be influenced by these potential moderators. Future research is recommended to explore this further, thereby clarifying the boundary conditions under which the model is applicable. (5) Due to the cross‐sectional design of this study, it is impossible to establish causal relationships between variables. Furthermore, the research focused solely on how occupational coping self‐efficacy mediates the relationship between authentic leadership and nurses’ caring behavior, without considering the influence of other confounding factors (such as organizational culture). Future studies should adopt longitudinal or experimental designs and incorporate diverse individual and organizational factors such as psychological empowerment and organizational climate. This would enable an exploration of the interactive mechanisms and influence networks from a dynamic perspective.

## 5. Conclusions

This study employs SEM to reveal that among nurses in Chinese tertiary hospitals, the promotional effect of authentic leadership on caring behavior is fully mediated through occupational coping self‐efficacy as a key psychological resource. This finding clarifies the core mediating role of occupational coping self‐efficacy, providing new empirical evidence for understanding the underlying psychological mechanisms through which authentic leadership influences nurses’ caring behavior. Nursing managers should actively demonstrate authentic leadership styles and implement strategies to enhance nurses’ occupational coping self‐efficacy, thereby strengthening their caring behavior. However, it must be noted that the cross‐sectional design limits causal inferences. Future studies may adopt longitudinal tracking or experimental designs to provide robust evidence for causal relationships among variables. Furthermore, future studies are encouraged to explore whether key sociodemographic factors (e.g., age, shift type, and work experience) moderate the relationships among authentic leadership, occupational coping self‐efficacy, and caring behavior. This could help identify specific groups of nurses who may benefit most from targeted interventions to enhance their occupational coping self‐efficacy.

## 6. Implication for Nursing Management

This study offers significant implications for nursing management practice. To effectively promote nurses’ caring behavior, nursing managers should actively practice authentic leadership styles, foster an open and trusting organizational atmosphere, and enhance nurses’ occupational coping self‐efficacy. Specific recommendations are as follows: First, senior management should promote systematic training in authentic leadership to help frontline nursing supervisors understand and practice authentic leadership behaviors such as self‐awareness and relational transparency. For example, leaders should share information with nurses, be receptive to different opinions and ideas, and provide timely feedback on nurses’ suggestions. Second, multiple approaches should be employed to enhance nurses’ occupational coping self‐efficacy. This can be achieved by conducting scenario‐based simulations, enabling nurses to accumulate successful experiences in clinically relevant challenges and bolster their confidence in handling situations. Organizing experience‐sharing activities encourages nurses to discuss typical cases or collective issues, fostering positive behaviors through vicarious learning. Implementing structured peer support programs establishes mechanisms for emotional mutual aid and professional feedback among colleagues.

## Funding

No funding was received for this research.

## Ethics Statement

The study was conducted according to the guidelines of the Declaration of Helsinki and approved by the ethics committee of Zhejiang Chinese Medical University (approved number: 20241108‐3).

## Consent

Informed consent was obtained from all subjects involved in the study.

## Conflicts of Interest

The authors declare no conflicts of interest.

## Data Availability

The data that support the findings of this study are available from the corresponding author upon reasonable request.
